# What Works for Older Adults to Reduce Problematic Alcohol Use: A Delphi Study on Elements and Mechanisms in Interventions

**DOI:** 10.1111/dar.70237

**Published:** 2026-09-08

**Authors:** Fieke A. E. van den Bulck, Andrea D. Rozema, Rob H. L. M. Bovens, Dike van de Mheen, Rik Crutzen

**Affiliations:** ^1^ Tranzo Scientific Center for Care and Wellbeing, School of Social and Behavioral Sciences Tilburg University Tilburg the Netherlands; ^2^ Positive Lifestyle Foundation Nijmegen the Netherlands; ^3^ Department of Health Promotion Care and Public Health Research Institute, Maastricht University Maastricht the Netherlands

**Keywords:** alcohol use, Delphi study, intervention, older adults, program theory

## Abstract

**Introduction:**

Understanding elements for older adults to reduce their problematic alcohol use is important for the development of interventions. This study aimed to establish expert consensus on effective and applicable elements, including their underlying mechanisms to reduce problematic alcohol use; and explore which elements are most effective and which mechanisms are important for older adults with lower‐ and higher‐severity problematic alcohol use, resulting in a programe theory.

**Methods:**

A three‐round questionnaire‐based Delphi study was conducted in the Netherlands. In the first (*n* = 20) and second round (*n* = 18), experts with treatment, research, policy, intervention development, educational and lived experience, rated 85 elements on their effectiveness and applicability, and mechanisms on exploratory power. In the third round (*n* = 18), 40 elements and 39 mechanisms with consensus were ranked on effectiveness and importance.

**Results:**

The most effective and applicable elements with consensus were *addressing age‐related issues* and the *combination of drinking and smoking*; *using motivational interviewing techniques*; having an *empathic, supportive and listening practitioner*; and *having therapeutic alliance.* Mechanisms best explaining the elements' effectiveness were *enabling early identification and timely intervention,* and *feeling recognised and acknowledged in group interventions*. For older adults with lower‐severity problematic alcohol use, insight into their use was particularly important, whereas for those with higher‐severity, self‐efficacy was important.

**Discussion and Conclusions:**

Elements and mechanisms related to motivation, personalisation, lifestyle, life stage, and relational connections with practitioners and/or group members were found particularly effective and important. The programe theory can inform the development of interventions for older adults with problematic alcohol use.

## 1 Introduction

The increasing number of older adults (55+ years) with problematic alcohol use calls for effective interventions and insight into what works for this demographic [1, 2, 3]. In the Netherlands, 5.4% of older adults engage in excessive drinking (≥ 21 drinks per week for men and ≥ 14 for women) and 5.3% of older adults engage in heavy drinking (≥ 6 drinks for men and ≥ 4 for women per occasion at least once per week) [4]. The proportion of older adults in Dutch addiction care for problematic alcohol use has increased substantially in recent years [5], while most interventions used for older adults are designed for the general adult population and less interventions are tailored to older adults [6, 7, 8]. Given the growing number of older adults, and their vulnerability to alcohol use due to age‐related physiological and metabolic changes and increased medication use [9, 10, 11], further investment is needed in what works specifically for older adults in interventions, ensuring these are effective for and applicable to this population.

There are a few studies that have examined what works in interventions for older adults to reduce problematic alcohol use, using a Realist Evaluation (RE) approach. RE examines how interventions and their elements, meaning the concrete components of an intervention that are delivered to participants, work differently across contexts. Interventions may be effective in some contexts but not in others because the mechanisms through which elements contribute to a successful intervention outcome are activated to a different extent [12]. The review by Boumans et al. [13] on interventions to reduce problematic alcohol use among older adults showed that of 61 included studies, only 3 evaluated interventions that were specifically designed for older adults. This review showed that providing information on the consequences of alcohol consumption, and providing personalised feedback about drinking behaviour in interventions helps to reduce problematic alcohol use among older adults [13]. An initial program theory was developed, combining findings specific to older adults with those concerning general adults. To further develop this initial program theory, three other studies also using an RE approach examined the perspectives of professionals, older adults and their relatives and identified together 53 elements and 21 mechanisms that contributed to reduced problematic alcohol use for this population in interventions in several contexts [14, 15, 16]. The interventions in these three studies focused on universal, selective or indicated prevention. They were provided in an individual and/or group setting with or without a relative's involvement, and were provided face to face, online and/or via telephone. Using a personalised approach, focusing on abstinence, receiving support and having contact with others seem important according to all perspectives. Differences between perspectives were that professionals mentioned the general mechanisms of ‘low threshold participation’ and ‘awareness’, while older adults highlighted ‘motivation’, and relatives mentioned having ‘control’ and a ‘deterrent’ to drink as mechanisms to reduce problematic alcohol use among older adults [14, 15, 16].

Building on the initial program theory, developed through the review [13] and perspectives from professionals, older adults and their relatives on elements and mechanisms in interventions for older adults to reduce problematic alcohol use [14, 15, 16], a next step is to establish consensus on what works best for older adults while considering two aspects. The first is to consider the applicability of the elements, as this is key to their usefulness and potential for successful implementation in interventions. The second is to take into account participant characteristics, as this is important to understanding what works for *whom*. For elements or needs in interventions, some studies look at gender within older adults [17, 18] or sexual identity within older adults [19], yet exploration of distinctions by other characteristics remains limited [6, 20, 21, 22]. In addition, a few studies highlighted variations in treatment barriers and engagement among older adults in relation to gender, marital status and social connectedness [18, 23, 24]. For example, loneliness, having contacts with healthcare providers, and not being married or partnered are positively associated with receiving treatment [23]. The severity of alcohol use may be another important aspect to understand for whom treatment barriers, engagement and intervention needs exist. For example, most adults seeking treatment seem to have a high severity of alcohol use disorder, while a larger group with a mild to moderate severity do not often seek treatment [25]. Moreover, those with higher severity compared to lower severity problematic alcohol use report a lower willingness to seek professional treatment if alcohol‐related problems occur, and they reported more barriers related to seeking treatment [26]. Similarly, severe substance use was associated with a greater perceived need for treatment, but also with more perceived barriers, particularly among older adults [27].

The aim of the current study was twofold: (i) to establish expert consensus on which elements are both effective and applicable to reduce (problematic) alcohol use among older adults, and to establish consensus on the mechanisms through which the elements contribute to their own effectiveness; (ii) based on the elements and mechanisms on which consensus was reached, we subsequently examined which elements are perceived as the most effective, and which mechanisms were considered most important, both for older adults with lower severity and for older adults with higher severity problematic alcohol use.

## 2 Method

### 2.1 Study Design

A three‐round Delphi study using questionnaires was conducted in the Netherlands to achieve the aim of this study. The Delphi method is used to establish experts' consensus through an iterative process that converges individual opinions towards a shared perspective [28, 29]. Participants rate items anonymously, for example, in surveys, and provide feedback on the group responses. This anonymous process allows participants to provide their opinions independently, while reducing the influence of other participants. In this study, the items evaluated were derived from the elements and mechanisms identified in the program theories developed in previous interview studies with Dutch professionals, older adults and their relatives [14, 15, 16]. As a result of the iterative process, the Delphi method enabled the participants to suggest additional elements and mechanisms and then to structure and refine them. Only elements and mechanisms that received high ratings and reached consensus were carried forward to the next round, as this allowed the prioritisation process to focus on elements and mechanisms with strong expert agreement regarding their effectiveness and importance. This study received approval from the Ethics Review Board (RP1411) of Tilburg University. All included participants read an informed consent form and gave their consent prior to the first Delphi round. The study was conducted and reported in accordance with the Conducting and Reporting Delphi Studies guidelines, see Supporting Information 1 [30].

### 2.2 Sampling and Recruitment

First, a list was made of aspects of expertise we considered important for participants in this study, including: (i) clinical, diagnostic, preventive or treatment experience with older adults with (problematic) alcohol use, or experience in coordinating or managing these; (ii) experience in developing or implementing interventions aimed at (problematic) alcohol use, such as prevention programs or treatment plans; (iii) experience in developing policies and guidelines aimed at reducing problematic alcohol use; (iv) experience in research focusing on older adults and/or problematic alcohol use; (v) experience in providing education, training and information to professionals to reduce problematic alcohol use; (vi) representatives of older adults, people with problematic alcohol use, and relatives of people with problematic alcohol use. Second, names of experts in the Netherlands were searched for in each area of expertise through a combination of the researchers' network and snowballing techniques, with a minimum of three per category, ensuring that all categories remained represented even in the case of dropouts during the rounds.

In total, 30 experts were sent an invitation and information letter about our study and the time frame for data collection. Six did not respond, two did not view themselves as experts, and two were unable to participate. All participants who completed the first round were invited to participate in the second and third round.

### 2.3 Data Collection and Analysis

#### 2.3.1 First Round: Elements and Mechanisms From Previous Studies

Experts were sent a link to the questionnaire of the first round via Qualtrics. The first round started with an informed consent procedure and demographics were assessed, that is, gender, age and background information about their job title, organisation and years of working experience in their field.

Participants were asked to rate the extent to which 53 elements are effective on a 6‐point Likert scale from 1 (‘definitely not’) to 6 (‘definitely’), with one extra answer option ‘I don't know’. These elements were organised by intervention context: in person or not; in group setting or individual setting, with or without practitioner involvement, with or without relative involvement. When an element was rated a 5 (‘very probably’) or a 6, we asked participants to rate the element on the same 6‐point Likert on applicability, and to rate the associated mechanism on a Likert scale from 1 to 6 for its explanatory power regarding its effectiveness. This explanatory power refers to the extent to which the underlying mechanism can explain the effectiveness of an element. When an associated mechanism was missing, that is, not identified in previous interview studies [14, 15, 16], an open‐ended question was posed to ask which mechanism ‘definitely’ explains the effectiveness of this specific element. Finally, at the end of this round, experts were asked whether there were elements not mentioned which they believed would be ‘definitely’ effective. We asked about the corresponding intervention contexts of each of these new elements.

The analysis of elements and mechanisms and the determination of their consensus among the experts was conducted according to a protocol defined a priori. Any element rated with a median score of ≥ 5 on effectiveness and applicability, combined with an interquartile range (IQR) of ≤ 1 or an overall minimum score of 4 (‘probably’) for effectiveness, was considered to be effective with consensus on its effectiveness. These elements were referred to as having ‘consensus in’. Any mechanism deriving from the interview study that was linked to a ‘consensus in’ element and rated with a median score of ≥ 5 on explanatory power for effectiveness, combined with an IQR of ≤ 1 or an overall minimum score of 4 (‘probably’) for explanatory power, was referred to as having ‘consensus in’.

Before the assessment of suggested new additional elements and new mechanisms could take place in the next round, the input provided by respondents was screened and organised by FB and RC. Suggested new elements were excluded if they were non‐intervention elements, conditions rather than elements, or mentioned as non‐effective elements. Suggested new mechanisms mentioned in the open mechanism question were grouped based on similarity.

#### 2.3.2 Second Round: New Elements and Mechanisms

This round contained the *new* elements and *new* mechanisms mentioned by experts in the first round. These elements were assessed in the same way as in the first round. Experts could only evaluate the mechanisms linked to elements they had individually rated with a score of ≥ 5 on effectiveness in the first round. The assessment of elements and mechanisms and the determination of their consensus was conducted according to the same a priori protocol as described in the first round.

#### 2.3.3 Third Round: Prioritising Elements and Mechanisms With Consensus

The aim of this third round was to prioritise the elements and mechanisms, on which consensus had been reached, for two groups: older adults with risky and excessive alcohol use (i.e., lower‐severity problematic alcohol use); and for older adults with severe problematic alcohol use and addiction (i.e., higher‐severity problematic alcohol use). The meaning of the terms ‘risky’, ‘excessive’, ‘severe problematic alcohol use’ and ‘addiction’ was not specified to the experts, because we did not want to impose this, but rather to rely on the experts' own interpretation. Also, we did not want to place undue emphasis on a specific classification logic, such as the level of consumption, negative consequences of alcohol use, or dependence. We considered that providing explicit explanations of these terms might have unintentionally guided experts' interpretations in order to answer the questions. Instead, we intended to emphasise a global distinction between less severe and more severe forms of problematic alcohol use. Forty elements and 39 mechanisms that were referred to as having ‘consensus in’ in the first and second round were divided over 6 element and 5 mechanism themes, inspired by those formed in the interview studies [14, 15, 16]. For each theme, we asked participants to rank half of the remaining elements or mechanisms, with a maximum of five items per theme. The reason for selecting half of the remaining items per theme, is that this was deemed the most efficient way to shorten the overall content (i.e., taking into account participant burden). Elements were ranked based on ‘most effective’, and mechanisms on ‘most important’ to reduce problematic alcohol use among older adults. Participants were asked to order each category by indicating which elements they placed as ‘1’, ‘2’, and so on. They were asked to complete these rankings for both groups.

For the third round, we assigned points based on the position each element and mechanism received in the participants' ratings. Each element received a score, with higher positions earning more points. For example, the theme ‘Paying attention to alcohol use’ had nine remaining elements, and we asked for a ranking of the four most effective. Then, each element was assigned points. If an item was ranked first, it received 4 points, second received 3 points, third received 2 points, and fourth received 1 point; if an item was not included in the ranking, it received zero points. These scores per element were then summed across all participants. For each theme, total scores of each element were analysed for a final ranking. The same method was applied to the mechanisms.

#### 2.3.4 Summarising the Delphi Rounds in a Program Theory

The first and second round identified effective elements and mechanisms, while the third revealed for whom these elements and mechanisms should be prioritised with regard to older adults with lower‐severity problematic alcohol use and older adults with higher‐severity problematic alcohol use. Using findings from all three rounds, we developed a refined program theory. This program theory explains, which effective and applicable elements contribute to reducing problematic alcohol use for older adults and links these elements to the mechanisms that explain their effectiveness. Therefore, to categorise the elements, we reformulated and rearranged the original themes from the previous interview studies [14, 15, 16], which were also used in the first and second round to provide a more specific reflection of the elements included in the program theory, in contrast to the broader themes previously used.

## 3 Results

In total, 20 participants completed the first round. The second round was completed by 18 participants, whereas a partly different group of 18 from the first round completed the third round. See Table 1 for an overview of participant characteristics per round.

**TABLE 1:** Characteristics of participants.

Participant characteristics	Round 1	Round 2	Round 3
	(*n* = 20)	(*n* = 18)	(*n* = 18)
Job title			
Addiction specialist	1	1	1
Advisor	2	1	0
Leadership or management role	4	3	4
Member of client council	2	2	2
Nurse specialist/community psychiatric nurse	2	2	2
Policy officer	2	2	2
Prevention specialist	6	6	6
Project worker	1	1	1
Professor	2	2	2
Psychiatrist	1	1	1
Expertise^b^			
Treatment and prevention, or coordination or management of these	11	10	10
Development or implementation of interventions	15	13	13
Development of policies and guidelines	6	6	6
Research	7	6	7
Provision of education, training and information to professionals	14	13	13
Representation of older adults, people with problematic alcohol use, and relatives of people with problematic alcohol use	4	4	4
Age, years			
30–54	10	9	9
≥ 55	10	9	9
Gender			
Woman	12	11	10
Man	8	7	8
Working experience with the topic of alcohol and/or older adults, years^a^			
1–10	8	7	6
11–20	3	2	3
21–30	5	5	5
31–45	3	3	3

^a^ One participant did not answer this question.

^b^ As participants were allowed to select more than one expertise category, the total exceeds the number of respondents.

### 3.1 Elements and Mechanisms With Consensus

An overview of the results of the first and second round can be found in Table 2. This table provides information about the median effectiveness, applicability and exploratory scores, their IQR scores, and whether all participants rated them between four and six. The elements with an effectiveness score of *M* = 6 are considered the most effective for reducing problematic alcohol use among older adults: *providing information and paying attention to age‐related issues* and to the *combination of drinking and smoking*; *using motivational interviewing techniques*; *having an empathic, supportive and listening practitioner*; and *having therapeutic alliance*. The mechanisms with an explanatory score of *M* = 6 are considered best explaining the effectiveness: *enabling early identification and timely intervention* and *feeling recognised and acknowledged* in group interventions.

**TABLE 2:** Ratings of elements and mechanisms.

Elements	Effectiveness		Applicability		Consensus	Mechanisms	Explanatory effectiveness		Consensus
	*M* (IQR)	Score 4–6	*M* (IQR)	Score 4–6			*M* (IQR)	Score 4–6	
Theme 1: Attention to alcohol use									
Having a conversation about alcohol use with practitioner	5 (0,25)	x	5 (1)		x	Reflecting on own behaviour*	5 (1)	x	x
						Enhancing motivation*	5 (1)	x	x
						Gaining insight and awareness*	5 (0)	x	x
						Creating openness*	4 (1)		
Paying attention to recognising problematic use	5 (1)		5 (0)	x	x	Enabling early detection and promoting earlier intervention*	6 (1)	x	x
						Gaining insight and awareness into own behaviour*	5 (1,5)	x	x
Looking at history of problematic use with practitioner	5 (1)		5 (1)	x	x	Gaining insight into the causes and underlying factors*	5 (1,5)	x	x
						Gaining insight into needs*	4 (1)	x	
Reflecting on alcohol use and cause of use with practitioner	5 (2)	x	6 (1)	x	x	Gaining insight into alcohol use	5 (0,75)		x
						Thinking about alcohol use	5,5 (1)	x	x
Temporary pausing of alcohol use*	5 (1)		5 (2)	x	x				
Receiving information about standard drinks*	5 (2)		6 (1)	x					
Tracking alcohol use	5 (0,25)		5 (1,5)	x	x	Thinking about alcohol use and gaining insight into it	5 (1)	x	x
Receiving information about how alcohol works in the body*	5 (2)		6 (1)	x					
Receiving information about alcohol, such as the possible effects and risks of alcohol use	4,5 (1)		6 (0,75)	x					
Having a conversation about complaints that are not perceived as alcohol‐related, but that may in fact be so*	5 (0,75)	x	6 (1)		x				
Discussing risk of relapse and regarding it as a normal part of the process*	5,5 (1)	x	6 (1)	x	x				
Learning to deal with cravings	5 (2)		6 (0,25)	x					
Paying attention to abstinence, practising abstinence and experiencing abstinence with health benefits	5 (1)	x	5 (1)	x	x	Breaking habits*	5 (1)	x	x
						Learning to deal with triggers*	5 (1)	x	x
						Learning that abstinence gives more peace of mind than moderating alcohol use*	5 (1)		x
						Learning new skills*	4,5 (1)	x	
						Enhancing motivation*	5 (1)	x	x
						Enhancing awareness*	5 (1,75)	x	x
						Enhancing confidence to successfully change alcohol use*	5 (1)	x	x
Doing daily breath alcohol tests with monitoring	3 (1,75)		5 (1)	x		Being deterred from drinking	4 (0)	x	
Having to account for alcohol consumption and abstinence	3 (1,5)		3 (0)						
Setting a daily abstinence goal, meaning the focus is not long‐term	4 (0)		5 (0,5)	x		Small goals make abstinence more achievable	5 (0,5)	x	
Theme 2: Attention to lifestyle and other									
Paying attention to meaning in life	5 (0,75)	x	6 (0)	x	x	Underlying cause of problem is eliminated*	4,5 (2)	x	
						Gaining insight into alternatives for alcohol use*	4 (1)		
						Being able to give life goals, direction and perspective*	5 (1)		x
						Enhancing motivation to change*	5 (1)		x
						Not feeling empty or lost*	4 (1)		
Paying attention to values that contribute to recovery*	5 (0,75)	x	5 (1)		x				
Discussing work or volunteer work*	4,5 (1)		5 (1)	x					
Sharing tips for activities, or engaging in activities together with peers	4,5 (1)		5 (0)	x					
Being offered alternative behaviours*	5 (1)	x	5 (1)	x	x				
Paying attention to grief and loss*	4,5 (2)	x	6 (2)	x					
Paying attention to age‐related issues	6 (1)	x	6 (0,25)	x	x	Creating a personalised approach*	5 (2)	x	x
						Being triggered*	4 (2)		
						Gaining motivation to change alcohol use*	4 (1)		
						Gaining awareness*	5 (2)		
						Gaining insight into relationship between age‐related problems and alcohol use*	5 (2)	x	x
Learning about lifestyle change	5 (1,25)		5 (1,75)	x					
Receiving information about alcohol use in combination with smoking*	6 (1,5)	x	6 (1)	x	x				
Theme 3: Approach and method									
*The intervention or practitioner is …*									
Using motivational interviewing techniques	6 (1)	x	6 (1)	x	x	Creating awareness	5 (1,25)	x	x
Using the social norms approach*	5 (0,5)	x	5 (1,5)	x	x				
Using cognitive behavioural therapy*	5 (2)	x	6 (1)	x	x				
Using the 12‐step Minnesota model	4 (1)		5 (0,5)	x		Gaining insight into change process	5 (0,5)	x	
Using reminiscence (recalling specific positive memories: vivid memories of a positive event on a certain day)*	5 (1)		4,5 (1,25)						
Using computerised cognitive bias modification through computerised interventions, aimed at automation of behavioural decisions*	4 (2)		5 (2)	x					
Using appropriate terminology: for example, not everyone with problematic alcohol use recognises themselves in the term ‘addiction care’*	5 (2)	x	5 (1,5)	x	x				
Using well‐coordinated network care*	5 (1)	x	4 (1,25)						
Providing a personalised approach, based on individual preferences and needs	5,5 (1)		5 (1)	x	x	Receiving personalised care	5 (1)	x	x
						Disappearance of non‐commitment	4 (2)		
						Receiving emotional support	4 (1)		
Aligning with client's needs*	5 (1)	x	5,5 (1)	x	x				
Expressing appreciation to the client*	5,5 (1)	x	6 (1)	x	x				
*The client is …*									
Participating in AA or other self‐help groups	4 (1)		5 (0,75)	x					
Mentioning positive experiences*	5 (1,75)	x	6 (1)	x	x				
Reducing the client's stigma*	5 (1,75)	x	4 (2,5)						
Receiving feedback from practitioner	5 (0,5)		6 (1)	x	x	Thinking about alcohol use*	4,5 (1)	x	
						Gaining insight and awareness into behaviour and alcohol use*	5 (1,5)	x	x
						Receiving alternatives for alcohol use*	4,5 (1)	x	
						Feeling rewarded by positive feedback*	5 (0)	x	x
Receiving a newsletter on a daily or regular basis	3 (2)		5 (0)	x		Regularity strengthens or changes perception of alcohol use	4 (0)	x	
						Remaining actively engaged with topic	4 (0)	x	
Having a role model or inspirational figure*	4 (1)		4 (2)						
Theme 4: Contact with practitioner									
*The practitioner …*									
Knows about client's history*	5 (2)	x	4 (0,5)						
Knows about client's culture*	5 (2)	x	5 (1)		x				
Is aware of complexity of older adults, shaped by their life stage, somatic conditions and alcohol use*	5 (1)		5 (1)	x	x				
Is aware of attitudes that may downplay alcohol use in older adults, such as assumptions like ‘they deserve their drink’*	5 (1)		5 (1)		x				
Has timely insight into cognitive disorders*	5 (1)	x	4 (1)						
Has a critical and confronting approach	4 (2)		5 (1)	x		Creating insight into the problems and need to change	5 (2)	x	x
Has an empathic, supportive and listening approach	6 (1)	x	6 (1)	x	x	Creating engagement and open communication between client and practitioner	5 (1)	x	x
Is a peer‐support practitioner	5 (1)	x	6 (1)	x	x	Feeling recognised and understood	5 (0,75)	x	x
Maps out client's social network*	5 (2)	x	4,5 (2)						
*Between the client and practitioner …*									
There is a therapeutic alliance*	6 (1)	x	5 (1)		x				
There is trust and connection for the client	5 (1)	x	5 (2)	x	x	Creating open communication between client and practitioner	5 (1,75)	x	x
The contact with the practitioner is accessible	5,5 (1)	x	5,5 (1)	x	x	Gaining trust in the relationship with practitioner, which creates receptiveness to practitioner's input*	5 (1)	x	x
						Feeling heard and seen*	5 (1)	x	x
						Gaining motivation*	5 (1)		x
						Feeling less hesitant*	5 (1)		xx
						Experiencing more openness in communication*	5 (0,75)		x
						Having low‐threshold contact*	5 (1)	x	x
The contact is on a regular basis	5 (2)		6 (1)	x					
The contact can take place at flexible times	4 (1,5)		4 (2)	x					
Theme 5: Contact in group									
*Presence of …*									
Group members with similar level of (problematic) alcohol use	5 (1,75)		4 (1)	x					
Group members with the same social status	3 (0,75)		5 (0)	x		Having a connection with group members	6 (0)	x	x
A group or discussion leader who monitors group dynamics and content	5 (1)	x	5 (1)	x	x	Having structure*	5 (2)	x	x
						Feeling safety and trust*	5 (0,25)	x	x
						Preventing a negative dynamic*	5 (1)	x	x
A closed group, meaning that there are no new members allowed, and there is a maximum group size	5 (1)		5 (1,5)	x	x	Becoming familiar with group members*	5 (0)	x	x
						Providing safety and trust*	5 (1)	x	x
*Group members are …*									
Able to reach one another at all times	4 (1)		5 (1)						
Participating in an introductory week prior to treatment program	4 (1)		4 (0)	x		Creating group cohesion	4 (0)	x	
Sharing experiences, choices and tips with group members	5 (1,5)		5 (2)						
Addressing one another regarding their behaviour and alcohol use	4 (1)		5,5 (1,75)	x					
Supporting one another and are understanding	5 (1,5)	x	5 (0,25)	x	x	Feeling recognised and validated*	6 (1)	x	x
						Gaining motivation*	5 (1)	x	x
						Feeling heard*	5 (0,25)	x	x
						Gaining trust*	5 (1)	x	x
						Feeling supported*	5,5 (1)	x	x
Having contact, openness, engagement and mutual respect in their relationship	5 (0,25)	x	5 (1)	x	x	Improving adherence to the intervention*	4 (1)		
						Gaining motivation*	5 (1)	x	x
						Creating safety and accessibility*	5 (1)	x	x
						Feeling heard and taken seriously*	5 (1)	x	x
*The client is …*									
Having online peer contact	4 (0)		4 (1)						
Experiencing group peer pressure*	4 (2)		4,5 (1,25)						
Theme 6: Contact with relatives									
Cooperation between relatives and practitioner	5 (1,25)	x	5 (1)	x	x	Providing social support and assistance*	5 (2)	x	x
						Enabling a joint approach*	5 (2)	x	x
Use of a triadic working method*	4 (1,5)		5 (2)	x					
*The relative/partner is …*									
Writing an impact letter describing the negative consequences of client's alcohol use	3,5 (1,25)		5 (0,5)	x		Being confronted with causes of use	4 (0)		
Monitoring client's abstinence at home	3 (1,25)		3 (0)			Preventing the client from drinking	3 (0)		
Learning to deal with alcohol use of client	5 (2)		5 (0,75)	x					
Learning to approach client positively	5 (1,5)	x	4 (1)	x					
Receiving information about intervention	5 (2)	x	5 (0,25)	x	x	Partner can better support client	5 (0,25)	x	x
*The client is …*									
Receiving support from relative	5 (1)	x	4 (1)						
Receiving support in coping with an environment where others drink (more)*	5 (1)		5 (1,5)	x	x				
Theme 7: Setting intervention									
*Participation is …*									
Anonymous to the client's environment, meaning that others do not know the client participates	4 (2,5)		5 (1)	x					
Anonymous	3 (1)		6 (0,25)	x					
*The intervention …*									
Is at a location nearby home	5 (1)		4 (1)						
Is at a familiar and trusted location	4 (0)		4,5 (0,5)	x					
Is online or via telephone	4 (0,5)		5 (2)						
Is at work	3 (1)		—	—					
Has a pleasant and relaxed atmosphere	5 (1)		5 (2)	x	x	Creating safety, which enables learning*	5 (1,5)	x	x
						Fostering openness and vulnerability*	5 (1)	x	x
						Enhancing therapeutic alliance*	5 (1)	x	x
						Enhancing motivation to continue attending the intervention*	5 (0,75)	x	x
*The client is …*									
Participating actively and seriously in intervention sessions	5 (0,25)	x	5 (1)	x	x	Creating safety*	4 (1)		
						Enhancing more motivation*	5 (1)	x	x
						Taking more responsibility*	5 (1)	x	x

Abbreviations: AA, Alcoholics Anonymous; IQR, interquartile range.

* These elements and mechanisms were proposed by participants in the first round and rated in the second round. Elements and mechanisms without an asterisk originate from the initial programe theory and were rated in the first round.

### 3.2 Prioritised Elements and Mechanisms

In Table 3, we present an overview of the elements ranked in the third round as most effective, and the mechanisms ranked as most important, separately for older adults with lower‐severity problematic alcohol use, and for older adults with higher‐severity problematic alcohol use. For each theme, the upper element is ranked by experts as the most effective, as it received the highest score in round 3 of all elements that had reached consensus after rounds 1 and 2. Thus, for the mechanisms, the first is considered the most important among the mechanisms in this theme according to the experts, followed by the second most important mechanism, and so forth. Table 3 shows that across the six themes of elements, older adults with lower‐severity and higher‐severity problematic alcohol use share the same element on four of the themes. These shared most effective elements were: *being offered alternative behaviours*; *having an empathic, supportive and listening practitioner*; *contact, openness, engagement and mutual respect* in the relationship with group members; and *cooperation between relatives and practitioner*. Additionally, two out of the five themes show an overlap in the mechanisms that were prioritised, which were *feeling recognised and acknowledged* in group interventions, and *being motivated to attend the intervention*.

**TABLE 3:** Ranking of elements and mechanisms by severity of problematic alcohol use.

Older adults with lower‐severity problematic alcohol use	Older adults with higher‐severity problematic alcohol use
Elements	
Theme 1: Attention to alcohol use	
1. Tracking alcohol use	1. Paying attention to abstinence, practising abstinence and experiencing its health benefits
2. Having a conversation about complaints that are not perceived by the client as alcohol‐related, but that may in fact be so	2. Reflecting on alcohol use and its underlying causes with the practitioner
3. Temporary pausing of alcohol use	3. Having a conversation about complaints that are not perceived by the client as alcohol‐related, but that may in fact be so
4. Paying attention to recognising problematic alcohol use	4. The risk of relapse is discussed and regarded as a normal part of the process
Theme 2: Attention to lifestyle and other	
1. Being offered alternative behaviours	1. Being offered alternative behaviours
2. Paying attention to age‐related issues	2. Paying attention to age‐related issues
Theme 3: Approach and method	
1. Conducting conversations using motivational interviewing techniques	1. Using a personalised approach based on individual preferences and needs
2. Using a personalised approach based on individual preferences and needs	2. Conducting conversations using motivational interviewing techniques
3. Expressing appreciation towards the client	3. Expressing appreciation towards the client
4. Pointing out positive experiences	4. Using cognitive behavioural therapy
Theme 4: Contact with practitioner	
1. The practitioner is empathic, offers support and listens to the client	1. The practitioner is empathic, offers support and listens to the client
2. The practitioner is aware of the complexity of older adults, shaped by their life stage, somatic conditions and alcohol use	2. The practitioner is aware of the complexity of older adults, shaped by their life stage, somatic conditions and alcohol use
3. The contact with the practitioner is accessible for the client	3. The practitioner is aware of attitudes that may downplay alcohol use in older adults, such as assumptions like “they deserve their drink”
4. The practitioner is aware of attitudes that may downplay alcohol use in older adults, such as assumptions like ‘they deserve their drink’	4. A sense of trust and connection with the practitioner
Theme 5: Contact in group	
1. Contact, openness, engagement and mutual respect in the relationship with group members	1. Contact, openness, engagement and mutual respect in the relationship with group members
2. Group members support one another and show understanding	2. Presence of a group or discussion leader who monitors group dynamics and content
Theme 6: Contact with relatives	
1. Cooperation between relatives and practitioner	1. Cooperation between relatives and practitioner
Mechanisms	
Theme 1: Mechanisms focused on cognitive processes, behavioural change and skills	
1. Breaking habits	1. Enhancing motivation
2. Gaining insights into your own alcohol use	2. Building confidence to successfully change alcohol use
3. Enhancing motivation	3. Resolving the underlying issue of the problematic use
4. Building confidence to successfully change alcohol use	4. Providing direction, perspective and purpose in life
5. Gaining insight into the relationship between (age‐related) problems and alcohol use	5. Breaking habits
Theme 2: Mechanisms in contact with practitioner	
1. Feeling heard and seen	1. Gaining trust in the relationship with practitioner
2. Gaining trust in the relationship with practitioner	2. Feeling heard and seen
3. Feeling engaged	3. Feeling engaged
Theme 3: Mechanisms in contact with group members	
1. Feeling recognised and acknowledged	1. Feeling recognised and acknowledged
2. Feeling supported	2. Feeling safe
3. Feeling a sense of trust	3. Feeling a sense of trust
4. Feeling safe	4. Feeling supported
Theme 4: Mechanisms in contact with relatives	
1. Having social support from relatives	1. Having a joint approach with relatives
Theme 5: Mechanisms in setting and content of intervention	
1. Having motivation to attend the intervention	1. Having the motivation to attend the intervention
2. Receiving a (more) personalised approach	2. Receiving a (more) personalised approach

*Note:* Within each theme, the first element represents the element that was ranked by experts as most effective. Mechanisms are listed in descending order of importance.

### 3.3 Program Theory of Effective Elements and Mechanisms for Older Adults

**FIGURE 1:**
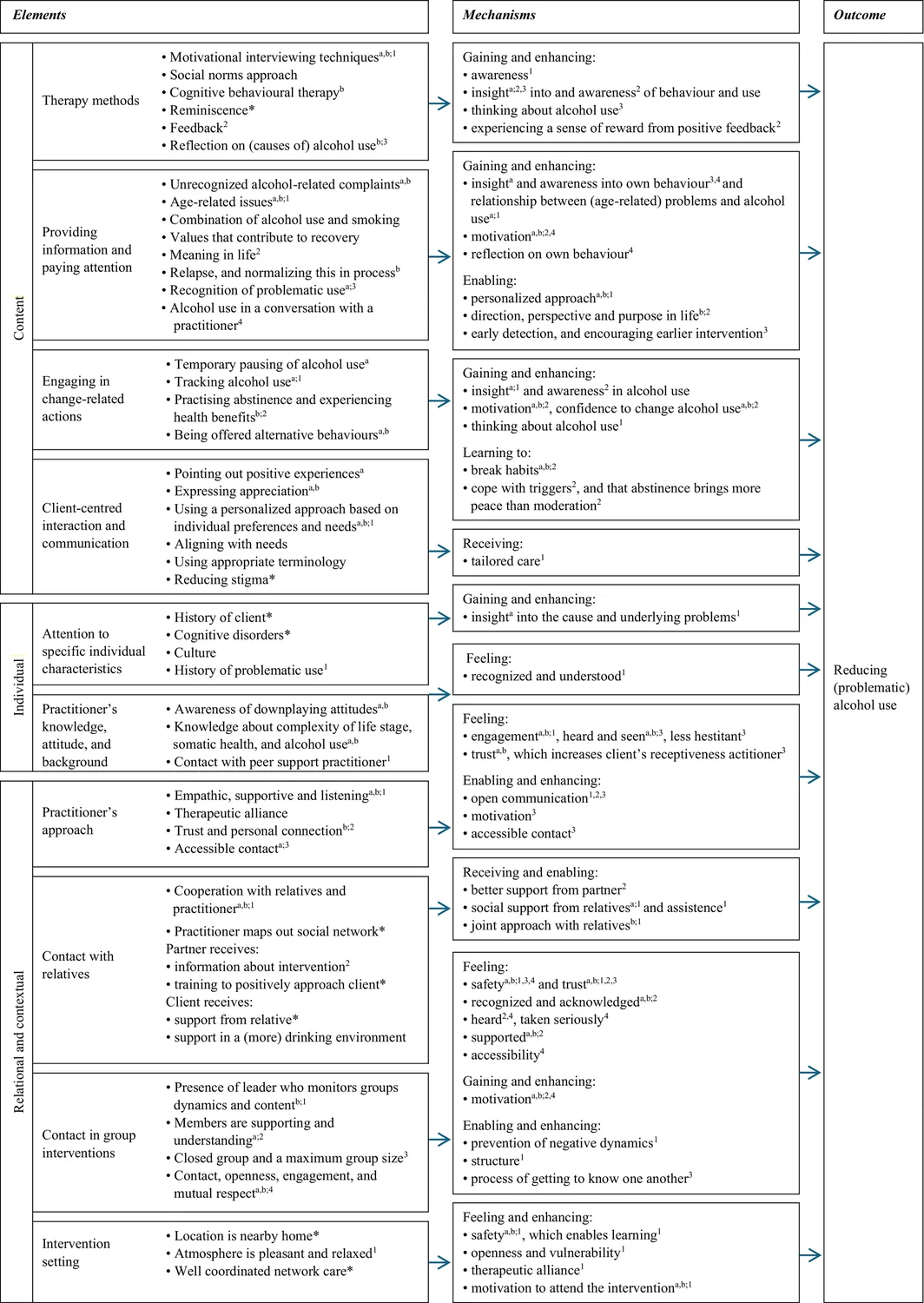
Program theory of effective elements and mechanisms for older adults in interventions to reduce problematic alcohol use. * = low applicability score. The superscript letters in elements indicate a high ranking of effectiveness within a specific target group. The superscript letters in mechanisms indicate a high ranking of importance within a specific target group. Here, ^a^ denotes that the element or mechanism was rated high for the target group of older adults with lower‐severity problematic alcohol use. Similarly, ᵇ refers to the high ranking of effectiveness for the target group of older adults with higher‐severity problematic alcohol use. The superscript numbers indicate the specific links between elements and mechanisms.

In Figure 1, we present the program theory with the effective elements and mechanisms with consensus after the first and second round, together with an indication of the elements and mechanisms that received a high ranking in the third round. Only 9 of 48 elements did not receive a high score on applicability. The elements can be divided into three categories. The first category consists of elements related to the intervention content: using therapy methods (*n* = 6); providing information about and paying attention to alcohol and lifestyle related matters (*n* = 8); letting the client engage in change‐related actions (*n* = 4); and using client‐centred interaction and communication (*n* = 6). The second category is comprised of elements indicating what needs to be addressed at the individual level: attention to specific individual characteristics of the client (*n* = 4); and the practitioner's knowledge, attitude and background (*n* = 3). The third category covers relational and contextual elements, addressing the clients' connections and the intervention's surrounding environment: practitioner's approach (*n* = 4); contact with relatives (*n* = 6); contact in group interventions (*n* = 4); and intervention setting (*n* = 3). Taken together, these findings—consisting of effective elements across 10 themes, their mechanisms and indications of which elements and mechanisms are particularly important for older adults with lower‐ and higher‐severity problematic alcohol use—form our final program theory.

## 4 Discussion

### 4.1 Key Findings

Of the 53 elements derived from the initial program theory, 24 were considered effective and applicable with consensus, while experts introduced an additional 16 effective and applicable elements with consensus. This resulted in a total of 40 elements included in our refined program theory, of which 11 were rated as highly effective both for older adults with lower‐ and those with higher‐severity problematic alcohol use: (i) having a conversation about unrecognised alcohol‐related complaints; (ii) being offered alternative behaviours; (iii) paying attention to age‐related issues; (iv) using motivational interviewing techniques; (v) using a personalised approach; (vi) expressing appreciation; (vii) the practitioner is empathic, supportive and listening; (viii) the practitioner has awareness of the complexity of life stage, somatic health and alcohol use; (ix) the practitioner has awareness of downplaying attitudes; (x) contact, openness, engagement and mutual respect in the relationship with group members (in group interventions); and (xi) cooperation between relatives and the practitioner.

The 21 mechanisms derived from the initial program theory, following their assessment and supplementation, resulted in a total of 39 mechanisms, of which a total of 12 were rated as highly important both for older adults with lower‐ and those with higher severity problematic alcohol use: (i) breaking habits; (ii) enhancing motivation; (iii) building confidence to successfully change alcohol use; (iv) having motivation to attend the intervention; (v) receiving a (more) personalised approach; and in contact with practitioner: (vi) feeling heard and seen; (vii) gaining trust in the relationship; (viii) feeling engaged; and in contact with group members in group interventions: (ix) feeling recognised and acknowledged; (x) feeling supported; (xi) feeling a sense of trust; and (xii) feeling safe.

Several elements and mechanisms can be considered generically effective and important, as they performed well across rounds and/or groups. We will first reflect on these findings, followed by a discussion of elements and mechanisms that were valued more specifically for one of the two groups.

### 4.2 Interpretation of Key Findings

#### 4.2.1 Generally Effective Elements and Important Mechanisms

First, among the generally effective elements, several related to lifestyle, that is, *being offered alternative behaviours* for alcohol use, and *paying attention to age‐related issues*. Older adults may face several challenges in physical or social areas (e.g., health problems and grief in later life) that can influence drinking behaviour [31, 32]. Moreover, lifestyle elements (e.g., physical activity and social participation) may help to reduce drinking [33]. However, for some older adults, alcohol use is embedded in daily and social routine, and greater social participation can even be linked to increased drinking [33, 34]. Here, the form of activities and the drinking context play an important role.

Second, several generally effective elements and important mechanisms were related to motivation and personalisation.

The element related to motivation was *using motivational interviewing techniques*, which is effective for older adults [14, 22, 35]. Moreover, being motivated to attend the intervention was considered highly important, as previously reported by older adults themselves [15]. In our study, this was linked to having *a pleasant and relaxed atmosphere* during the intervention. Because older adults are, compared to younger adults, generally less likely to seek help for alcohol use problems [27], it is also important to explore how to encourage them to participate. For brief alcohol interventions, informal community venue settings, for example, supermarkets, shopping centres, hospital atriums and health centres, appear acceptable among older adults for raising awareness of lower‐risk drinking, particularly for those not reached in traditional healthcare settings and not in need of intensive treatment [36]. When looking at more severe problematic alcohol use among adults, stigma is often a barrier to seeking help [37]. However, contact with healthcare providers has been linked to the initiation of treatment for older adults with alcohol use disorder [23].

The element related to personalisation referred *to a personalised approach based on individual preferences and needs*. Effective forms of such personalised approaches include the use of personalised conversations, feedback and reports [6, 7, 13, 14]. In our study, personalisation was also described as a mechanism of change, though that mechanism was linked to only one specific element: *attention to age‐related issues*. Other studies also highlighted the importance of age‐based tailoring, for instance by incorporating age‐tailored health messages [36, 38], and by adapting both content and structure to the needs of older adults, for example, including flexible delivery modalities (e.g., telehealth, in‐home services), creating a slower pace of learning, providing shorter sessions, and accommodating vision, hearing and cognitive impairments [39]. Additionally, gender is also considered an important factor around which to personalise treatment. For example, some older men primarily express a need for a non‐belittling approach and being heard, while some older women tend to prefer to be taught how to manage emotions [17]. Although age and gender can be relevant factors for tailoring personalisation, some studies suggest that preferred treatments were those tailored for the individual, rather than general factors such as age or gender [18]. These findings highlight the potential need for practitioners to balance demographic tailoring with a sensitivity to individual needs and preferences among older adults.

Third, several generally effective elements and important mechanisms were related to a relational connection between the client and both the group members in group interventions and the practitioner, as well as to the knowledge and attitudes of the practitioner.

In relation to group members, trust was an important mechanism, and *recognition and acknowledgement, feeling supported* and *a sense of safety* were important elements. These elements are also deemed important by professionals, older adults and their relatives [14, 15, 16]. With regard to the practitioner, it was important to have an *empathic approach*, to ensure that the client feels heard and seen, to build trust in the interaction, and to foster a sense of engagement. Also older adults with substance use problems prefer interventions that are led by warm, honest, caring and relatively non‐confrontational providers [40]. However, therapeutic contact with emotional attention, such as a warm and genuine therapist stance alone, may be enough for positive intervention outcomes, and older adults tend to do better when more directive elements are also included in the approach [41].

In addition, elements regarding the knowledge and attitudes of the practitioner including their awareness of *the complexity of older adults, shaped by their life stage, somatic conditions and alcohol use* and being aware of *attitudes that may downplay alcohol use in older adults*. This emphasises the need to balance general knowledge about this demographic, while also being aware of their own attitudes and biases towards this age group. A previous study showed that practitioners may sometimes hold negative views, perceiving older adults as difficult and dependent, which may contribute to unequal care provision [42]. Strengthening knowledge about age‐related issues, combined with a reflective and person‐centred approach, may help to foster engagement and improve care for older adults [42].

#### 4.2.2 Group‐Specific Effective Elements and Important Mechanisms

We found differences in prioritisation in elements focused on attention for alcohol use, and in mechanisms in cognitive processes, behaviour change and skills.

Among older adults with lower‐severity problematic alcohol use, several prioritised elements and mechanisms were focused on the client gaining insights. For instance, the elements *tracking alcohol use* and *paying attention to recognising problematic alcohol use* were associated with mechanisms of increasing insights into alcohol use. Moreover, two of the important mechanisms specifically for this group were also about gaining insights. A possible explanation for this is that people with lower‐severity alcohol use may not yet be fully aware of the problematic aspect of their drinking, as they may experience fewer alcohol‐related risks than those with higher‐severity use. Therefore, enhancing insight and awareness may serve as a first step towards change.

Among older adults with higher‐severity problematic alcohol use, we specifically observed elements and mechanisms related to enhancing self‐efficacy. Two important mechanisms in this group were increasing motivation and building confidence to successfully change alcohol use. People with higher‐severity problematic alcohol use may at times feel more discouraged or powerless to change their alcohol use, which makes self‐efficacy particularly important, including in preventing and dealing with relapse. One effective element for this group in the current study was the normalisation of relapse, that is, *the risk of relapse is discussed and regarded as a normal part of the process*. Strengthening self‐efficacy was also linked to *practising with abstinence*, which helped reinforce both motivation and confidence to maintain change in alcohol use.

### 4.3 Implications

This study contributes to the literature by identifying a set of elements that can be applied across a wide range of interventions aimed at problematic alcohol use among older adults, rather than being restricted to one specific intervention. As the applicability of the elements in this study was assessed within the Dutch context, two aspects of their applicability for intervention design beyond this context require further reflection.

Within the Dutch context, some elements were rated as effective but not easily applicable. First, we observed this in elements involving relatives, namely: *the practitioner maps out the client's social network*, *the partner learns to approach the client positively*, and *the client receives support from relatives*. The importance of being in contact and receiving support from relatives is also highlighted by older adults and their relatives [15, 16]. However, there may be barriers in involving relatives, namely organisational barriers (e.g., lack of funding and time), family‐related barriers (e.g., limited supportive networks) or personal barriers for the practitioner (e.g., lack of self‐efficacy to do family work, or misconceptions about the role of families) [43]. Second, we observed low‐applicability rates in elements related to the practitioner's knowledge of the client, namely: *reminiscence is applied*; *the practitioner knows the client's history*; and *the practitioner has timely insight into cognitive impairments*. Gaining a thorough understanding of the client's medical and social history requires time that may not be available within the scope of a typical care session. Finally, we observed low‐applicability rates in a few other elements; *reducing stigma for the client*; *the intervention takes place at a location close to home*; and *there is a well‐coordinated network care*. In order for interventions to use these elements that have a lower applicability score, more research is needed on the conditions of their implementation in interventions.

Although a few effective elements received lower applicability scores, most identified effective elements were considered applicable, suggesting they have promising implications for Dutch interventions. For professionals, it is important to be aware of this range of elements that can be used across different intervention settings, as some elements are for group interventions, interventions with the involvement of relatives or rely on direct practitioner–client interaction, while others are not specifically linked to any of these settings.

For the applicability of the elements beyond the Dutch context, it is important to reflect on them more broadly. Elements directly related to the biological aspects of ageing are likely to be universally applicable for older adults. Several other effective elements align with intervention elements reported in previous studies [8, 13], most of which were conducted in Western countries. However, differences exist across Western countries in alcohol use and drinking patterns among older adults, which are related to population composition and country‐specific contextual factors [44]. Some elements and mechanisms that were directly linked to someone's personal alcohol use may therefore differ in their prioritisation and application across contexts. Other elements that were linked to lifestyle and life stage may also be more context‐dependent, for example, dependent on the social norms and retirement regulations in one country. For example, when retirement is accompanied by more leisure time and opportunities for socialisation, it may lead to increased alcohol use [33]. It is therefore important to consider how the transition into later life is experienced, as well as the meaning and function of alcohol use in older adults' personal life. This points to the importance of personal relevance and tailoring interventions to the older adults' individual meaning of alcohol use [8].

### 4.4 Strengths and Limitations

A first strength is the use of the Delphi method, which enabled an iterative process through which a shared perspective was established among experts. A second strength of this study is the involvement of 18 to 20 experts with diverse expertise in alcohol use and older adults, which is a considerable number given the niche nature of the topic in the Dutch context. A third strength is our layered process in which the program theory was created. The elements and mechanisms were assessed by diverse perspectives and validated through input at several levels in addition to the earlier assessments by professionals, older adults and relatives.

In addition to its strengths, this study also has some limitations. The first is that the differences in the severity of the problematic alcohol use were only examined in the third round. Perhaps certain elements might have been considered as effective with consensus for one group, yet they did not make it through the first and second round. As a result, elements that may be beneficial for specific groups might not be sufficiently reflected in the overall findings. Accordingly, the program theory does not draw group‐specific conclusions about what was perceived as effective for whom. However, it provides additional indications of which subgroups of older adults with lower‐ and higher‐severity problematic alcohol use may benefit more from elements that were perceived as effective for a broader group of older adults. The second limitation is that due to the broad scope of what we aimed to assess, we had to deviate from the traditional Delphi approach, which led us to exclude elements with lower scores on effectiveness and/or without consensus from further analysis. As a result, it remains unclear whether lower effectiveness scores reflect ineffectiveness or are due to other influencing factors—for example, the underrepresentation of client and relative perspectives among included experts—since most lower‐scoring elements were primarily mentioned by older adults and relatives in previous studies [15, 16]. Therefore, some of them could nonetheless be important for clients' experiences, engagement or treatment preferences.

## 5 Conclusion

Elements and mechanisms related to motivation, personalisation, lifestyle and life stage, and relational connections with practitioners and/or group members are perceived as particularly effective in supporting older adults in reducing or stopping alcohol use and can be applied in interventions. Specifically, for older adults with lower‐severity problematic alcohol use, insight into their use is crucial, whereas those with higher‐severity require greater emphasis on self‐efficacy. The support and involvement of relatives seem promising for older adults, although further research is needed on its successful application in interventions. This study identified what works and why it works for older adults to reduce problematic alcohol use and adds to the literature a program theory specific to older adults that can support the development of interventions and provide practical guidance for practitioners who work with this population.

## Author Contributions

Conceptualisation: Fieke A.E. van den Bulck, Andrea D. Rozema, Rob H.L.M Bovens, Dike van de Mheen, Rik Crutzen; Methodology: Fieke A.E. van den Bulck, Andrea D. Rozema, Rob H.L.M. Bovens, Dike van de Mheen, Rik Crutzen; Formal analysis and investigation: Fieke A.E. van den Bulck, Rik Crutzen; Writing – original draft preparation: Fieke A.E. van den Bulck; Writing – review and editing: Andrea D. Rozema, Rob H.L.M. Bovens, Dike van de Mheen, Rik Crutzen; Funding acquisition: Andrea D. Rozema; Supervision: Andrea D. Rozema, Rob H.L.M. Bovens, Dike van de Mheen, Rik Crutzen.

## Funding

This work was supported by the Netherlands Organisation for Health Research and Development (ZonMw number: 555002034).

## Conflicts of Interest

The authors declare no conflicts of interest.

## Supporting information


**Supporting Information: 1:** Supporting Information.

## Data Availability

Research data are not shared.
